# Exploration of the Socioecological Determinants of Hong Kong Workers’ Work-Life Balance: A Grounded Theory Model

**DOI:** 10.3390/ijerph182010732

**Published:** 2021-10-13

**Authors:** Ka Po Wong, Alan Hoi Shou Chan

**Affiliations:** Department of Advanced Design and Systems Engineering, City University of Hong Kong, Hong Kong, China; alan.chan@cityu.edu.hk

**Keywords:** work-life balance, grounded theory model, personal context, physical and mental states, qualitative research

## Abstract

Background: Although a growing body of research has investigated the theoretical and empirical models of work-life balance (WLB), the propositions of this phenomenon remain nonunified. Thus, a grounded theory approach was adopted to explore the viewpoints of workers regarding WLB and its determinants and consequences. Methods: Individual face-to-face interviews were conducted to investigate the attitudes and experiences of Hong Kong workers towards WLB, in which 50 workers were interviewed. All data of interviews were transcribed verbatim and coded into five levels of the socioecological framework (i.e., intrapersonal level, interpersonal level, organisational level, community and government policy). Results: The grounded theory model established that work-life balance and personal context mutually affected each other, and work-life balance was unidirectionally affected by the environmental context. The ability to maintain a continual satisfied physical and mental states among multiple roles under the emergence of unexpected environmental factors was proposed as the definition of work-life balance. Conclusions: The findings of this study offer essential research insights into the importance of WLB, the dynamic features for workers to sustain balance and constructing a reliable and exhaustive assessment model for work-life balance for future studies.

## 1. Introduction

Work-life balance has always been a topical issue in workplaces. Given the pressing demands for work-life balance practices, such as flexible work hours, various initiatives have been formulated to address the reasonable needs of employees. However, some practices are incomplete, and some are merely ostentation. Thus, employees achieving a healthy work-life balance from their workplace is unlikely to be possible. Many researchers have attempted to define the concept of work-life balance and conducted empirical studies to assess the relatedness of antecedents and consequences with work-life balance. Practically, a new shift has taken place in the ways in which workers live and work. The needs of employees are varying with the changing socioeconomic environment; younger generations are entering the labour market; most of their lifestyle is shifting towards pursuing mental health rather than material aspects. Thus, an update of the current aspects related to work-life balance to recognise any change is imperative.

To fill the research gap, a qualitative method using the grounded theory approach was adopted in this study to identify how employees define work-life balance and the determinants that presently affect the perceived work-life balance of employees. A socioecological framework was adopted to develop a grounded theory model. The socioecological system was extensively used to comprehend the dynamic interrelationship among different personal and environmental conditions [1]. The factors influencing the changes in personal behaviours should be understood to serve as guidance in assisting in the formulation of appropriate programs in certain communities and environments. In comparison with traditional theoretical approaches to work-life balance, the novel socioecological system could certainly be used to explore the phenomenon of work-life balance. This study allows researchers to understand how current employees perceive their work-life balance, and enable practitioners to execute efficacious strategies to mitigate obstacles. The research contribution is to construct an updated and thorough grounded theory model for work-life balance by analysing and compartmentalising the extracted data.

### 1.1. Initiation and Development of Work-Life Balance

Numerous conceptual definitions have been proposed for work-life balance since the early 2000s [2]. Casper et al. [3] classified these conceptual definitions into eight main categories: conflict, enrichment, satisfaction, effectiveness, equality, involvement, importance, and fit. Work-family conflict was the initial interest among researchers, rather than work-life balance [4,5,6]. Some researchers have recognised that the interaction between work and family domains may not only result in detrimental effects to individuals and the related parties, but may also offer advantages [7]. Thus, work-family enrichment, which refers to the mutual benefits in resources, personality development and performance between two roles, was suggested [8,9]. Greenhaus and Allen [10] conducted an extensive review on work-family balance in which the meaning, antecedents and consequences of work-family balance were identified. However, the investigation on the work-family interface is considered to be rather narrow. A harmonious relationship between the roles of work and life is upheld, considering that those without children do not place the family in an important position [11]. Life might include caring activities, community activities, hobbies and exercise. Some researchers have upheld the definition of Frone [12] that balance is minimising conflict between work and life domains. Others have advocated well-balanced satisfaction among different life spheres [2,13]. Hill et al. [14] proposed the conceptual definition of effectiveness for work-life balance, which is the degree to which an individual successfully balances multiple roles and performance. Some researchers have regarded balance as the equivalency of time involvement and resource allocation in multiple roles [15]. Therefore, the scales for work-life balance used in empirical studies are different. Such scales include perceived satisfaction on role balance [16,17], sufficient time allocation to each role [18], the value of different roles, and the adequacy of organisational practices [19]. An ongoing debate exists as to how work-life balance should be defined.

Despite the massive empirical studies conducted on work-life balance, the assessment on the factors and effects pertinent to work-life balance has been referred to studies that investigate the conflict and enrichment of work and life domains [20] or self-unearthed issues related to work-life balance [21,22,23]. Carlson et al. [24] found that the variances of role balance were greater than those of role conflict and enrichment, which implies that more facets can be explored from role balance. Despite the opposing views on the definition of balance, the purpose of work-life balance, which is to enhance individual wellbeing, remains consistent. Many scholars have indicated a spectrum of factors that affect individual work-life balance. Work-related factors include career objectives and development, cooperation with colleagues, job characteristics, relationship with and supports from managers and workplace policies and facilities [25,26,27,28,29]. Personal life-related antecedents include daily life habits, family context (e.g., family demand, involvement, relationship and support), interpersonal relationships and leisure activities [20,30,31,32,33]. Evidence shows that work-life balance has a positive effect on job performance, job satisfaction, organisational commitment, life satisfaction, quality of life and maintaining health [13,25,28,34,35,36,37].

### 1.2. Work-Life Balance in Hong Kong

Overtime work is not uncommon in Asian countries, as performing well at work is a strong culture in these countries, and many workers desire to fulfil the rising job demands [38]. This phenomenon causes chronic exhaustion and even incidents of Karoshi [39], and appears to confirm that workers in Asia have an unhealthy work-life balance. Cultural factors, which are significant factors impacting workers’ work-life balance, include the importance of social networks in Asian work values, collectivism and low gender egalitarianism. Social networking is an influencing factor leading to blurring boundaries between work and nonwork life in Asian countries [40]. The power of social networks is a significant work value in Asia, particularly in China, because Asian employees believe that strong social networks can boost career development [40]. Collectivist cultures emphasise the goals of a group instead of the desires of an individual, and group loyalty is upheld [41]. People are pushed by not wanting to let the group down. Therefore, workers are inclined to comply with the orders of management staff who possibly give high work demands to workers, resulting in work-life imbalance [42]. In addition, males are characterised as the breadwinners in many Asian countries [43]. The caring and family tasks are considered to be the responsibilities of women. Female workers may have to deal with family related matters in business time, possibly contributing to an imbalance between work and life roles [44].

Under the influence of Asian work values and cultures, workers in Hong Kong very commonly encounter long working hours [45,46]. The workers probably are required to handle work-related tasks in private time, receiving work-related calls or emails when on holiday or after work since the expectation of employers is that they will be available at any time. As a result, a healthy work-life balance is an unattainable goal for many Hong Kong workers. Hong Kong workers have a worse work-life balance when compared with many other large cities, and Hong Kong has been ranked as the most overworked city [47,48]. Although the Hong Kong SAR government has promoted family-friendly policies, the voluntary basis of the setting may fail to reflect the effectiveness of such policies. The survey results about the work-life balance of Hong Kong workers published by Community Business in 2014 showed that nearly half of the participants were unable to achieve a healthy work-life balance, and that the result had remained almost unchanged for nine years (i.e., 2006 to 2014) [49]. This result implies that there was inadequate provision of support to assist workers in improving their work-life balance. A great emphasis is placed on work-life balance rather than material returns. Most workers prefer more flexible working hours and locations to raises. Workers proactively attempt to change their current working conditions to achieve work-life balance. However, without the support of the government and organisations, it is difficult for workers to attain work-life balance [50]. Furthermore, the survey also found that males, white-collar workers and respondents with a higher educational level had a higher degree of achieving ideal work-life balance than their counterparts did. These findings indicate that the perceived work-life balance varies among workers. A public opinion programme of work-life balance survey of Hong Kong employees is conducted by the University of Hong Kong every year, the questions of which are about organisational culture with respect to work-life balance, office location, and facilities inside and outside the workplace. The questions of this survey mainly focus on work domain, and nonwork aspects have been practically overlooked. The focus of the survey is excessively narrow, and it cannot thoroughly evaluate the work-life balance of employees in Hong Kong.

### 1.3. Aims

This study aims to explore the socio-ecological determinants of work-life balance among Hong Kong workers using a grounded theory approach. While Hong Kong is a metropolitan city mixing Asian and western cultures, its work culture is steeped in Chinese traditions and cultural norms. The working hours in Hong Kong are notoriously demanding, leading to a great deal of work stress among workers. Meanwhile, the socioecological system can demonstrate the complex interrelationship between individual and environmental factors, and this system will be adopted in this study. Furthermore, the grounded theory approach allows us to achieve a deep understanding of the phenomenon and build a new theory. Therefore, the work-life balance of Hong Kong workers is worthy of being investigated in order to identify the major socioecological factors affecting the work-life balance of the workers and formulate a new theory of work-life balance, particularly for fast-paced cities.

## 2. Materials and Methods

Individual face-to-face interviews were used in this study to collect and analyse qualitative data. Attitudes, experiences and views related to work-life balance of individual participants were explored in the interviews, the relationships of which were identified and categorised [51,52]. Given the appearance of new and unpredictable information and knowledge in data analysis, theoretical sampling was employed [53]. This information is important in conducting coding analysis to construct a grounded theory model. Theoretical preconceptions were avoided in the data analysis. Grounded theory has been widely applied in different research areas, such as clinical [54,55], management [56] and death research [57].

### 2.1. Data Source

The interview questions were compiled on the basis of prior studies, including the antecedents [58,59,60] and outcomes [37,61,62] of work-life balance. The questions aimed to explore how the participants perceived the meaning of work and personal life; identify the factors affecting the work and personal life roles and the balance between these roles; and determine the outcome of work-life balance. In addition to the demographic information, the interview questions were mainly classified into the following categories: (1) general understanding of work, life and work-life balance; (2) factors affecting work-life balance at work; (3) factors affecting work-life balance in personal life; (4) outcomes of work-life balance; and (5) reactions to work-life imbalance. The semi-structured interview, including principal questions and follow-up questions if necessary, was prepared to obtain relevant information on work-life balance. Examples of questions in each category are as follows:

(1) General understanding of work-life balance:

- In your opinion, what is life?

- In your opinion, what is work?

(2) Factors affecting work-life balance at work:

- At work, what are the factors affecting your attitudes and performance towards work?

- Does your company have policies or measures related to work-life balance?

(3) Factors affecting work-life balance in personal life:

- If you can do more important things or activities you previously mentioned, how will your work-life balance be affected?

- If you can do more necessary but unimportant things or activities you previously mentioned, how will your work-life balance be affected?

(4) Outcomes of work-life balance and work-life imbalance:

- What do you think is the effect of a healthy work-life balance on your work?

- What do you think is the effect of an unhealthy work-life balance on your daily life?

(5) Reactions to work-life imbalance

- What do you do when faced with work-life imbalance?

### 2.2. Data Collection Procedures

All interview procedures were approved by the Human Subject Ethics Subcommittee of the authors’ university. The participants were recruited in Hong Kong. The eligibility criteria for the participants were as follows: (1) aged 18 and above; (2) currently salaried employees; (3) working at least one year; and (4) can speak Cantonese. People who were interested contacted the first author, who conducted a brief screening to determine eligibility. One week was given to the qualified participants to consider whether to participate in the interview. The participants were allowed to read the consent conditions and ask the first author about this interview and research. Once the consent forms had been agreed to and signed, it was arranged for the participants to partake in the individual interview. A, HKD 50 cash coupon for a local supermarket was given to the participants as remuneration. Each interview was approximately 45–60 min, in which the participants could casually discuss their opinions and views regarding the phenomenon of work-life balance.

### 2.3. Participants

A total of 50 participants (21 females and 29 males) were recruited for the study. The sample size met the minimum requirement of 15–30 samples suggested by Flick et al. [63]. The age ranges of participants were 18–24 (6%), 25–34 (36%), 35–44 (30%), 45–54 (18%) and 55–64 (10%). The educational level of the participants ranged from the completion of junior middle school to the completion of a doctorate degree. A total of 52% participants had earned bachelor’s degrees or above. A total of 48 participants were full-time employees, and two participants were part-time employees. The majority of the participants were single (40%). A total of 28% of participants were married with child/children, and 32% participants were married without children. For average monthly income, the three ranges referred to the 2019 Pay Trend Survey of civil service pay level. A total of 40% participants estimated less than $22,865, 52% participants estimated $22,865–70,090, 6% participants estimated $70,090–140,560 and one participant did not respond. Fifteen types of industry were involved. 

### 2.4. Data Analysis

The audio of interviews was transcribed verbatim, and the data were analysed by the qualitative data analysis application software Nvivo 12 Pro. This analysis method involved a three-stage coding process [64]. Open coding, the first phase, progressively examined the verbatim data [53]. The data were segmented, detected and categorised to identify different phenomena and obtain the clues [53]. Codes were clearly identified, such that the direct reactions and attitudes of the interviewees towards a phenomenon were constituted [65]. Axial coding, known as theoretical coding, was the second stage of the data analysis. It involved the combination of categories and subcategories. The obviously related concepts were combined, and unique categories based on each related coding for conducting comparison and explaining the phenomenon were constructed [66]. The processes of data collection, interpretation and induction were repeated until the categories and subcategories were saturated or no new data were revealed [64]. In selective coding, which was the last stage, a core category was constructed to link all categories [53]. The core category was used to reflect the relationships among different categories and explain the most fundamental phenomenon [53].

## 3. Results

The analysis of the data showed three main aspects related to work-life balance: (1) definition of work-life balance; (2) determinants affecting work-life balance in the socioecological framework; and (3) the development of a theoretical model for identifying causal conditions, facilitators and barriers of work-life balance.

### 3.1. Definition of Work-Life Balance

One of the concerns of this study was how employees defined work-life balance. A total of 92 quotes were coded, which were classified into seven categories: 1) able to do what I like/want to do after work (40.2%); 2) appropriate involvement in each role based on myself (22.8%); 3) defined by the duration of each domain (12.0%); 4) emotional states (12.0%); 5) meaningfulness of work (8.7%); 6) having money (2.2%); and 7) high flexibility between work and personal life domains (2.2%). This finding indicates that employees emphasise their autonomy and freedom in nonworking hours on the premise of getting off work on time.

### 3.2. Determinants Affecting Work-Life Balance in the Socioecological Framework

The results demonstrated different levels of socioecological determinants of work-life balance. These determinants emphasised by the participants are summarised in Table 1. The greatest mutual influencing factor for perceived work-life balance among participants was physical and mental wellbeing.

On the intrapersonal level, most participants believed and experienced that not suffering from any health problems, having sufficient and quality sleep, healthy lifestyles and positive attitudes towards various aspects of life could enhance work-life balance.

‘If I am suffering from headache, I will have less time to spend on my social life, losing the balance.’

‘If you are not feeling well, you can neither do what you want to nor do your work. Then, your balance will be affected.’

‘Work-life imbalance leads to irregular mealtime or skipping meals, resulting in insufficient intake of energy.’

Some participants stated that their work performance, emotions and feelings towards work, ability to deal with difficulties at work and getting career promotion could influence the level of their work-life balance. Moreover, the autonomy of the time after work and arranging quality time for leisure, social activities and personal matters were important elements for achieving work-life balance.

On the interpersonal level, most participants felt that their boss and colleagues could strongly affect them in maintaining a healthy work-life balance, especially their working attitudes and behaviours. The workload given by their boss and their relationship with their boss and colleagues were one of the significant determinants affecting work-life balance.

‘When my boss is highly involved in his job, I will be willing to spend more time on work to help him. But my overtime hours become longer.’

‘If the boss leaves work on time, my colleagues and I will not prefer to stay at office to overwork.’

‘If the boss is terrible and mean, then we will not exert additional effort on their work and we will feel stressed, which adversely affects the balance.’

Some workers noted that their relationship with their family members and peers could affect the balance between work and personal life roles. Several participants indicated that support from their family and friends could improve their work-life balance.

In terms of organisational factors, all participants claimed that the implementation of workplace policies could assist in achieving work-life balance. Annual leave was the workplace policy of most concern among the participants, with 25 participants stating that increasing annual leave could help achieve work-life balance.

‘I think the most important work-life balance initiative is increasing annual leave because only 10 days or so for annual leave is too few.’

‘Increasing annual leave will be likely to promote work-life balance.’

Certain participants mentioned that the establishment of work-life balance culture in the organisations, provision of opportunities for career advancement, and allowing autonomy and flexibility over their jobs could heighten the balance level.

For the influence of community, overtime working is a ubiquitous phenomenon in Hong Kong, and is becoming a social norm. Most workers noted that working overtime for one to two hours was acceptable. High social costs have led to the unpreventable working of overtime to earn a living. In social media at present, many people enjoy sharing where they go and what they eat. Some respondents compared themselves with others by questioning why others could spend so much time travelling.

Regarding the effects of government policies, some workers thought that implementing policies related to the safeguard of work-life balance of employees could certainly improve the balance level. Government policies influenced the operation of the organisations, affecting the workload and pressure on the employees. Furthermore, several participants mentioned that ill-judged administrative policies might trigger social movements that would affect emotions, work schedules and personal activities, thereby causing work-life imbalance.

### 3.3. Development of a Theoretical Model for Work-Life Balance

**Figure 1:**
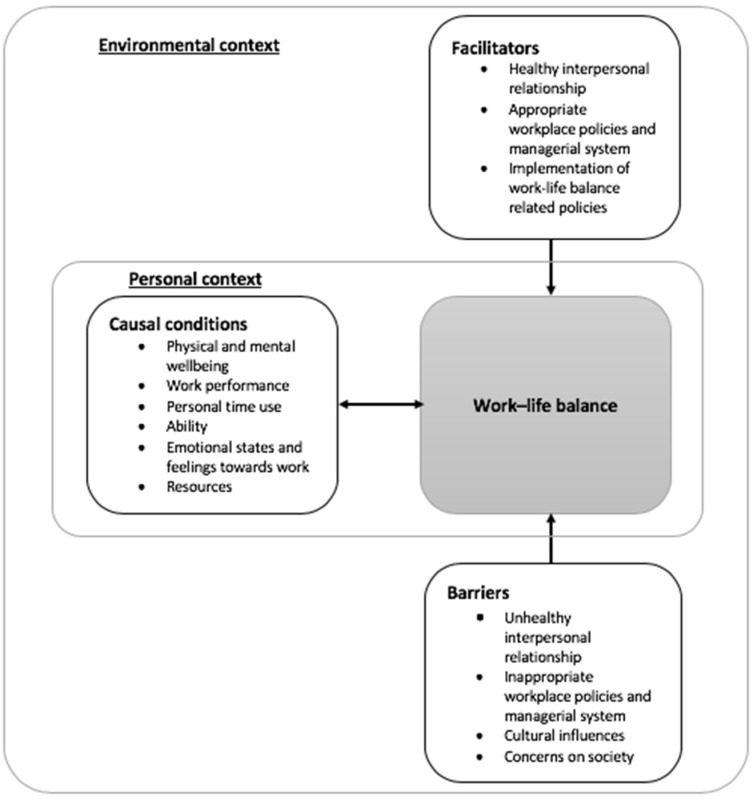
Grounded theory model for work-life balance.

Further analysis of the socioecological determinants of work-life balance led to the establishment of grounded theory model depicting the causal conditions, facilitators and barriers of work-life balance, which were classified into two context levels (Figure 1). The first layer was the personal context in which causal conditions were involved. The determinants at the intrapersonal level (e.g., physical and mental wellbeing, ability and emotional states at work) were the major contributors affecting the level of work-life balance of individuals. Participants’ responses revealed that physical and mental wellbeing; personal time use; work performance; and emotions and feeling towards work, ability and resources were affected by different levels of work-life balance. Thus, the personal context mutually influenced work-life balance.

‘If balance is achieved, my bodily condition will be healthy and I will be happy as well.’

‘Having fewer illnesses results in fewer worries, better mental health and a relatively lower chance of suffering from mood disorder.’

‘Having work-life imbalance, I will feel great pressure, which will lead to different diseases.’

In addition to the intrapersonal-level determinants directly affecting the level of work-life balance, the determinants in interpersonal and organisational levels and the factors related to community and government policies, which influenced the perceived work-life balance to some extent, served as the facilitators and barriers and generated the second layer—environmental context. The influences on the level of work-life balance possibly relied on how the workers became conscious of the external, sudden and unexpected events. The level of perceived work-life balance might fluctuate day by day as the attitudes and behaviours of workers would dynamically alter to react with these events, which were considered the facilitators and barriers. When encountering work-life imbalance, some workers preferred positive approaches, such as doing interesting activities, communicating with others, and rearranging their priorities, whereas some would disregard and tolerate it. A proposed definition of WLB is a subjective appraisal of the ability to maintain continually satisfying physical and mental states in multiple roles, and when subject to the emergence of unexpected environmental factors.

## 4. Discussion

In this study, the five levels of determinants within the socioecological framework were found to influence the level of work-life balance of the workers. The most important determinants affecting work-life balance were physical and mental wellbeing (e.g., bodily conditions, mental health, emotions and lifestyles). Other determinants were work performance, personal time use, ability, emotional states and feelings towards work and resources. A new grounded theory model generated from the socioecological framework included two layers, namely, personal and environmental contexts. Personal context constituted causal conditions, namely, intrapersonal-level determinants and environmental context composed of facilitators and barriers. The facilitators for maintaining work-life balance included healthy interpersonal relationships, establishment of appropriate workplace policies and managerial systems, and employee-friendly government policies. The barriers hindering workers in achieving work-life balance included unhealthy interpersonal relationships, inappropriate workplace policies and managerial systems, cultural influences, concerns regarding society, and social movements. Moreover, the meaning of work-life balance to most workers was autonomy in nonworking hours.

The findings regarding the meaning of work-life balance exhibited discrepancies with respect to previous definitions. Contrary to our expectations, most participants (40.2%) thought that with work-life balance, they could do what they would like to do after work, which was distinct from past definitions of work-life balance. However, some participants defined work-life balance in a similar way to how previous researchers did, including appropriate time allocation [67], being contented and delighted in certain states [12,68], and flexibility between roles [69]. The statement, ‘Being able to do what I like/want to do after work’, implied that the work domain is separate from the personal life domain, and they had no freedom in the time domain. This finding also differs from some published studies stating that work-life balance interfered, and that a spillover relationship existed between work and nonwork domains [8,12]. Employees in Hong Kong enjoy absolute autonomy in nonworking hours, which should be completely segregated from the work role. The working hours in Hong Kong are extremely long, leading to a relatively short length of personal time for workers. Thus, most Hong Kong workers value their private time and autonomy.

The intrapersonal-level determinants explored in this study, including physical and mental wellbeing, work performance, personal time use, and ability and emotions towards work, were comparable to the determinants of work-life balance in previous studies [18,25,36,60,61]. However, to the best of the authors’ knowledge, studies have rarely investigated whether personal feelings (i.e., happiness and job satisfaction) and economic gains were significant factors of work-life balance. Generally, researchers have been inclined to hypothesise that personal feelings are the outcomes of work-life balance. The findings regarding the interpersonal-level determinants (e.g., relationship with boss, colleagues, family and peers, support from family and friends, and feedback from customers) were in line with some previously identified work-related predictors of work-life balance [20,70,71]. Nevertheless, working attitudes and characteristics related to the boss and colleagues (e.g., efficiency, hardworking level and seriousness) affecting work-life balance, which have been seldom mentioned in previous studies, accounted for the greatest percentage of determinants regarding the boss and colleagues. A possible explanation for this result may be that the mixing of eastern and western cultures in Hong Kong contributes to relationships with coworkers being less important than how they perform at work. Most studies in this field have emphasised the importance of the support from supervisors and colleagues rather than their working attitudes and behaviours. Organisational determinants (i.e., workplace policies and managerial systems) as one of the factors influencing work-life balance corroborates the results of some previous work [22,26,72], in which workplace arrangements regarding breastfeeding support, dependent care, flexible working and work leave improved the work-life balance of employees. The effect of the community, including the social norm of long working hours, social needs, and the effects of social media, is in accordance with previous results indicating that long working hours are the norm among employees in Hong Kong [60]. Thus, most employees accept overtime work. This phenomenon is in opposition to the typical norms that overtime work adversely affects the work-life balance of workers, and that workers do not prefer to work overtime. The findings regarding the effects of social media on the perceived work-life balance are in agreement with the study of Aravinda Kumar and Priyadarshini [73], who found a significant influence of the use of social media on worker behaviours and work-life balance. The influences of policies, found in this study to be one of the determinants affecting the work-life balance of workers, support previous findings that family-friendly policies established by the government have a significant effect on work-life balance [19,74].

The workers described that their level of work-life balance would spontaneously change due to the sudden emergence of facilitators and barriers. Thus, they frequently strived to achieve a balance among multiple roles with respect to their determination and encountering facilitators and barriers. However, most studies demonstrated the determinants of work-life balance to be stiff and did not present the dynamic nature of workers making decisions by adjusting their attitudes and behaviours in the context of the advent of facilitators and barriers. These subconscious decisions regarding the maintenance of balance were reflected on wellbeing, emotions, feelings, ability and performance. Therefore, a mutually influential relationship was suggested regarding the interaction between personal context and work-life balance. This condition should be considered when assisting workers in achieving a healthy work-life balance.

Notably, some determinants for work-life balance and the findings in this study are similar to previous conceptual and theoretical frameworks related to work-life balance. Haar et al. [75] demonstrated a work-life balance model based on the demand and resource approach proposed by Voydanoff [76]. In the demand and resource approach, demands were classified into work demands (e.g., working hours and overtime work) and family demands (e.g., family size and parental status), and resources (i.e., supervisor support and job autonomy) were moderators of the relationship between work-life balance and demands. Husin et al. [61] suggested that family context, work context and flexible hours affected work-life balance, and that health factors served as a mediator. Lee and Sirgy [5] developed a work-life balance framework by synthesising previous studies about work-life interface. In the work-life balance framework, the factors affecting work-life balance were classified into personal and organisational predictors. The organisational predictors included the factors related to job characteristics and workplace policies for work-life balance. The grounded theory model used in the present study uses a socioecological framework instead of the traditional categorisations for work-life balance. In comparison with previous theoretical frameworks for work-life balance, the socioecological framework approach provides an unambiguous structure regarding the dynamic interplay between human behaviours and social and environmental factors. This approach also helps decision makers in obtaining a profound and precise understanding of the dynamic changes of the psychological status and behaviours of workers reacting to the influences of various external factors. This dynamic interaction has seldom been identified in previous research.

### 4.1. Theoretical Implication

This study utilised numerous significant research avenues and updated the new aspects pertinent to work-life balance. Firstly, physical and mental wellbeing was found to be the major influencing factor in the process of workers achieving work-life balance, whereas most previous studies tended to highlight work-related factors (i.e., duration of working hours, job autonomy and flexible work arrangement), which conflicted with the nonwork domain and jeopardised balance. The changes in physical and mental wellbeing were a straightforward response to external effects. For example, a heavy workload might influence the bedtime, emotions and psychological health of workers, meaning that their work-life balance might be affected. Thus, physical and mental wellbeing must be considered as the predominant determinants for work-life balance. Further research should be conducted to validate this proposition. This issue is important for future research. Secondly, the personality and working attitudes of the boss and colleagues seem to indirectly influence the work-life balance of employees. Previous studies have mainly focused on the effects of support and leadership of bosses or supervisors on maintaining work-life balance [36]. Further research should be conducted to determine this proposition. Thirdly, peer influences have been overlooked in previous works. The universality of the Internet and social media allows people to access others’ information easily, inviting comparisons of lifestyles with others and thus affecting perceived work-life balance. This hypothesis has to be further scrutinised for validation. Fourthly, the social norm regarding working overtime leads some employees to accept working beyond their scheduled working hours. This phenomenon should also be further investigated before validation. Lastly, financial wellbeing might be one of the important issues related to the ability of workers to achieve balance. When confronting unstable or insufficient earnings, balance might become meaningless to certain workers. Resolving the struggle between work-life balance and stable finances remains a conundrum. Further progress could be achieved by exploring how work-life balance is affected by financial wellbeing.

### 4.2. Practical Implications

Organisations and human resources practitioners can take advantage of the information provided by this study as a reference in determining fitting intervention strategies for assisting employees in attaining WLB. Human resource practitioners need to understand how WLB can be achieved; for instance, they should determine the main causes, facilitators and barriers for workers. Physical and mental wellbeing should be the focus in WLB policies. Many Hong Kong workers emphasise autonomy in personal time on the premise of getting off work on time. Since technologies enable people to connect at any time, supervisors or management staff should avoid the temptation to contact their subordinates outside working hours. Furthermore, most Hong Kong workers believed that to improve WLB, it is necessary to increase the length of annual leave and to arrange leisure activities and cultivate hobbies. Taking breaks from work can ease negative emotions and feelings towards workplaces and supervisors and help to regain perspective on work. Broadly, improving WLB will be advantageous for both employees and employers because it elevates the wellbeing of workers, lowers absenteeism and increases productivity. To implement these initiatives effectively, communication between supervisors and subordinates must be enhanced, and thus appropriate resources can be arranged for employees.

### 4.3. Limitations

Several important limitations must be considered. Firstly, the cautiousness of interviewees in revealing details might be a restriction, because some sensitive information regarding the work and personal life of the participants was involved in the interviews. To make the participants feel comfortable in expressing their feelings and views, the entire process of interviews was performed using a casual and informal approach, and it was promised that all information would be kept confidential. However, obscure expressions from interviewees continued to be observed throughout the interviews. Secondly, the data of this study have to be investigated using quantitative methods. The significance and the weightings of the identified antecedents and outcomes need to be identified because the data were subjective. Quantitative methods allowed the research gap to be filled, and a validated work-life balance model could be constructed. Thirdly, although the cultural, environmental and economic realities of Hong Kong are different from those in other regions or countries, the findings of this study have referential importance regarding the handling of issues of role balance. Lastly, the limitations with respect to time and resources might be a constraint. Some participants might not be willing to spend time (i.e., more than 60 min) on an interview. Thus, its completeness might be affected.

## 5. Conclusions

This study aimed to comprehensively investigate the perceived work-life balance of employees in Hong Kong using a qualitative approach. A socioecological framework and a grounded theory were developed regarding work-life balance. Five levels of determinants related to work-life balance were identified in the socioecological framework, namely, intrapersonal level, interpersonal level, organisational level, community, and government policy. In the grounded theory model, the socioecological determinants, which were categorised into causal conditions, facilitators and barriers, formed two layers: personal and environmental contexts. An interaction with a dynamic nature was found between the attitudes and behaviours of workers perceived work-life balance and environmental factors. The results of this study provide new theoretical insights into the dynamic influential relationship between work-life balance and individual context affected by environmental context. Recommendations for organisations and employers are provided to formulate appropriate policies for assisting employees in achieving work-life balance.

## Figures and Tables

**Table 1:** Description of socioecological determinants affecting work-life balance (The total number of quotes was 1264.).

Level	Summary of Quotes
Intrapersonal (65.11%)	**Physical and mental wellbeing (73.51%)**
	Experiences of physical and mental health problems (83.21%)
	Sleep quality and sufficiency (7.77%)
	Positiveness, meaningfulness and level of enjoyment (6.12%)
	Being able to exercise (2.15%)
	Having regular and quality meals (1.16%)
	Disregard or tolerance of work-life imbalance (0.50%)
	**Work performance (8.99%)**
	Work efficiency, productivity and quality (74.32%)
	Concentrated at work, confidence at work and responsibility for work (25.68%)
	**Personal time use (8.26%)**
	Arranging time for leisure and social activities (77.94)
	Being able to do what they like to do after work (14.71%)
	Quality and quantity time for doing personal matters (7.35%)
	**Ability (4.13%)**
	Dealing with urgent or challenging job tasks (70.59%)
	Career path and promotion (14.71%)
	Time management (6.06%)
	Pursuing further study for career development (5.88%)
	**Emotional states and feelings towards work (4.74%)**
	Job satisfaction, happiness at work, passion at work, positivity at work, sense of accomplishment, sense of belonging and willingness to accept tasks (76.92%)
	Work involvement and engagement (12.82%)
	Turnover intention (10.26%)
	**Resources (0.36%)**
	Economic losses/gains (100%)
Interpersonal (20.89%)	**Boss and colleagues (58.71%)**
	Working attitudes and characteristics of the boss and colleagues (35.58%)
	Workload (e.g., working overtime, working at home and working during nonoffice hours) assigned or expected by the boss (27.10%)
	Relationships with boss and colleagues (20.65%)
	Cooperation with colleagues (11.61%)
	Recognition and appreciation by the boss and manager (5.16%)
	**Family (29.17%)**
	Relationship with family members (44.29%)
	Quality time for accompanying family (32.86%)
	Support from family (15.71%)
	Financial burden from family (7.14%)
	**Peer (10.23%)**
	Relationship with friends (55.56%)
	Comparisons with friends on working attitudes and living quality (33.33%)
	Support from friends (11.11%)
	**Customers (1.89%)**
	Attitudes, communications and feedback of customers (100.00%)
Organisational (12.10%)	**Workplace policies (94.77%)**
	Increasing annual leave; compensation for overtime work; establishment of maximum working hours and five working days per week; flexible working hours; home offices; increase in labour; leaving work on time; provision of facilities for dependent care, meals and rest; provision of welfare on transport; and proper work leave arrangement (100.00%)
	**Managerial system (5.23%)**
	Organisational culture on work-life balance (37.50%)
	Career advancement (25.00%)
	Chance for employees to control over their jobs (25.00%)
	Allowing flexibility for employees (12.50%)
Community (1.27%)	**Cultural influence (81.25%)**
	Social norm of long working hours (69.23%)
	Social needs (23.08%)
	Effects of social media (7.69%)
	**Concerns on society (18.75%)**
	Concern or awareness on the social issues (100%)
Government policy (0.63%)	**Government policies (100%)**
	Implementation of policies related to work-life balance (12.50%)
	Social movements caused by the dissatisfied administrative politics (87.50%)

Note: Determinants are in bold.

## Data Availability

The data presented in this study are available on request from the corresponding author. The data are not publicly available due to their containing information that could compromise the privacy of research participants.
